# Multiplexed coding through synchronous and asynchronous spiking

**DOI:** 10.1186/1471-2202-16-S1-P198

**Published:** 2015-12-18

**Authors:** Milad Lankarany, Steven A Prescott

**Affiliations:** 1 Neurosciences and Mental Health, The Hospital for Sick Children, Toronto, ON, Canada; 2 Department of Physiology and Institute of Biomaterials and Biomedical Engineering, University of Toronto, Toronto, ON, Canada

## 

The prodigious capacity of our brain to process information relies on efficient neural coding strategies. In engineered systems, bandwidth is often increased through multiplexing - multiple signals are simultaneously, yet independently, transmitted through a single communication channel. We have proposed previously that neural systems might implement the same sort of solution [1]. Here, we tested if/how multiplexed coding could be achieved through combined rate and temporal coding. We hypothesized that a set of neurons could independently encode two signals by using asynchronous spike rate to encode one signal and synchronous spike timing to encode the other.

**Figure 1:**
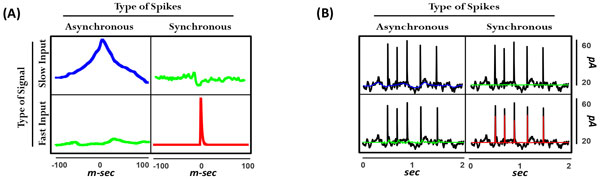
**Multiplexed coding (A) STAs calculated from slow or fast signals using asynchronous or syncrhonous spikes**. (**B**) Reconstructed inputs (colored) using two classes of spikes and corresponding STAs versus original stimulus (black).

To test our hypothesis, we built a feed-forward neural network comprising Morris-Lecar (ML) model neurons. All neurons received a common input constructed from two distinct signals, slow and fast, plus uncorrelated fast noise. According to our hypothesis, slow and fast signals are independently encoded by different types of spikes; in other words, differentially correlated output spikes, namely, asynchronous (Async) and synchronous (Sync), enable encoding of slow and fast signals, respectively. To assess the feasibility of the multiplexed coding scheme, recorded spikes were classified into two independent classes based on the peristimulus time histogram (PSTH) calculated from the entire set of neurons. Spikes whose instantaneous rates exceeded a threshold were designated "Sync" and all others were designated "Async". The spike triggered average (STA) was calculated for slow and fast signals using Sync and Async spikes, resulting in four different STAs. The Async-slow and Sync-fast STAs were clearly structured whereas the other two were not (Figure 1A). Using the two spike types to calculate STAs from the single combined fast-slow input gave two similarly structured STAs (meaning that one does not need *a priori *knowledge of which signal evokes which spikes) whereas treating all spikes as equivalent compromised STA measurement, especially for the fast signal (meaning that one needs to subdivide spikes according to their level of synchrony). Beyond simply measuring STAs, Figure 1B shows that convolving the appropriately measured STA with the synchronous or asynchronous spike train enables excellent reconstruction of the fast and slow signal, respectively.

Our results demonstrate the feasibility of multiplexed coding using synchronous and asynchronous spiking. Decoding the two signals requires that spikes are distinguished by their cross-correlation; this is difficult to do with experimental data in which only a subset of neurons are recorded, but it is straightforward for the brain using downstream neurons that operate as coincidence detectors or integrators that respond preferentially depending on input correlation.
